# Effects of Acibenzolar-S-methyl on the Probing Behaviour and Mortality of *Cacopsylla pyri* on Pear Plants

**DOI:** 10.3390/insects13060525

**Published:** 2022-06-06

**Authors:** Stefano Civolani, Daniele Mirandola, Lorenzo Benetti, Luca Finetti, Marco Pezzi, Giovanni Bernacchia

**Affiliations:** 1 Department of Environmental Sciences and Prevention, University of Ferrara, Via Borsari 46, 44121 Ferrara, Italy; cvlsfn@unife.it; 2 Innovaricerca Srl, Via Pastorella 17, 44124 Ferrara, Italy; 3 Department of Life Sciences and Biotechnology, University of Ferrara, Via Borsari 46, 44121 Ferrara, Italy; mrndnl1@unife.it (D.M.); lorenzo.benetti@edu.unife.it (L.B.); pzzmrc2@unife.it (M.P.); 4 Department of Biology, University of Toronto Mississauga, 3359 Mississauga Road, Mississauga, ON L5L 1C6, Canada; luca.finetti@utoronto.ca

**Keywords:** pear psylla, acibenzolar-S-methyl, chemical elicitor, PR protein, EPG system

## Abstract

**Simple Summary:**

Acibenzolar-S-methyl is an analogue of salicylic acid, and it is known as a plant elicitor able to induce plant endogenous defences against plant pathogens. Recently, it has been shown to also affect phloem-feeder pests, even though the mechanism is still unclear. Pear psylla (Cacopsylla pyri) is a serious threat for pear production in Europe, and its control is usually based on the use of chemical insecticides. The development of novel innovative control approaches is becoming more and more important, especially in integrated pest management. The present work investigated the possible indirect influence of acibenzolar-S-methyl, through the expression of pear Pathogenesis-Related protein (PR) coding genes, on the probing behaviour and on the survival of C. pyri nymphs and adults feeding on pear potted plants. The minor effects observed on the pest would suggest that acibenzolar-S-methyl cannot be used against psyllas, but it might be recommended on pear orchards in the primary control of other targets such as fire blight disease.

**Abstract:**

European pear psylla, *Cacopsylla pyri*, is one of the worst pests of pear in Europe. We investigated whether acibenzolar-S-methyl (ASM) application on pear plants might affect the behaviour in *C. pyri*. The elicitor was applied on pear potted plants, and after 48 h, we confirmed the ASM-mediated induction of several Pathogenesis-Related protein (*PR*) coding genes. At the same time, an in-depth analysis was performed on the probing behaviour of adults and nymphs of *C. pyri* on ASM-treated pear plants by the EPG-DC system, as well as the assessment of young nymphs’ survival 7 days after the ASM application. The elicitor application weakly interfered with *C. pyri* nymphs probing behaviour and survival, while it did not affect adult stages. These data confirm previous observations obtained on *C. pyricola* and suggest that the elicitor does not represent a viable tool in the control of pear psylla species, especially if used alone, but it might be used in integrated management strategies focused on other plant pathogens such as *Erwinia amylovora*.

## 1. Introduction

The pear psylla *Cacopsylla pyri* L. (Hemiptera: Psyllidae) is one of the most important pests of the common pear (*Pyrus communis* L.) across Europe [1]. The damage is mainly due to the honeydew excreted by nymphs, which harms leaf tissues and creates fruits russeting. Honeydew also acts as an optimal growth medium for black sooty moulds, whose presence on fruits drastically reduces their market value [2]. In addition, *C. pyri* is a vector for “*Candidatus* Phytoplasma pyri” responsible for the Pear Decline disease (PD) [3], a severe condition that reduces tree vigour and may even be lethal for trees.

During the last two decades the control programs based on integrated pest management (IPM) were able to obtain great results in containing *C. pyri* damage in European pear orchards. This IPM strategy was based on the initial use of conventional insecticides (abamectin and spirotetramat) followed by the natural control exerted by enemies such as the predator *Anthocoris nemoralis*, unaffected by the chemicals used. The chemical management of the psyllid proves to be very effective because of very low pest resistance toward the active ingredients [4,5,6].

In the last few years, the excessive application of nonselective insecticides to control the massive invasion of orchards by the alien marmorated stink bug, *Halyomorpha halys*, has induced a decrease in *C. pyri* natural predators thus triggering delayed near-harvest outbreaks that have often caused significant damage to pear production [7]. On the other hand, abamectin is soon to be banned in the European Union, further limiting the number of products that can be used in the orchard. Therefore, new tools need to be developed in the near future.

A promising strategy to control *C. pyri* involves the host plant’s innate resistance mechanisms [8]. Nin and colleagues maintain that all main *P. communis* cultivars are still susceptible to all pear psylla species and despite the long-term worldwide breeding programs, no commercially resistant cultivar has been released.

Conversely, some researchers have suggested the use of plant resistance elicitors, such as harpins, chitosans, and acibenzolar-S-methyl (ASM), to control *C. pyricola* [9,10,11]. Among these compounds, the ASM, a synthetic analogue of salicylic acid (SA), has no direct effect against pests but causes the activation and accumulation of several pest-resistant proteins and molecules in the treated plants [12,13,14]. Among these, several PR (pathogenesis-related) proteins, upregulated by ASM treatment, show different chemical and physical properties such as chitinase (PR3 and PR8), glucanase (PR2), and antimicrobial activities (antifungal PR1 and osmotin/thaumatin-like PR5). [15,16].

ASM has been successfully applied against pear diseases including fire blight, caused by *Erwinia amylovora*, due to its ability to induce Systemic Acquired Resistance (SAR). Therefore, it has been registered for foliar application to control fire blight and other pear pathogens [17,18,19,20]. The possibility of using ASM to activate plant defences against phytophagous insects has proved uncertain [21,22]. On one hand, ASM was shown to induce defence responses against the phloem-feeding green peach aphid, *Myzus persicae*, on tomato [23,24,25]. On the other hand, Cooper and Horton [9,10] have shown that ASM application might affect congeneric *C. pyricola* nymphs’ infestations in the northeastern USA, although they did not provide evidence of the plant responses activated by the elicitor and did not suggest a possible mechanism of action that might explain how this elicitor affected the pests. Furthermore, Orpet and colleagues [11] reported that treating pear plants with harpin or ASM did not cause a long-term induction of the PR1 gene or a reduction in the *C. pyricola* population.

Our study aims to investigate whether short-term ASM treatment (as verified by induction of several defence genes) can interfere with *C. pyri* probing behaviour and nymphs’ survival in pear plants.

## 2. Materials and Methods

### 2.1. Source of Insects and Plant Material

All *C. pyri* adult winter forms were field collected and directly used for EPG-DC recording. Otherwise, reared *C. pyri* nymph and adult summer forms were maintained on 1-year old potted pear plants (30 cm height) of the susceptible cv. Abbé Fétel and kept in a climate-controlled chamber at 23 ± 1 °C under a 16:8 LD photoperiod.

### 2.2. ASM Applications

A commercial solution of ASM (Bion^®^ 50 WG; Syngenta Crop Protection, Milan, Italy) was dissolved in distilled water. The concentration applied was 125 mg L^−1^. One year old potted plants of susceptible cultivar Abbé Fétel were randomly assigned to treatment and removed from the greenhouse prior to solution application in an open field by hand atomizers. Plants were sprayed until runoff of leaves, left to dry for 1 h, and then returned to the greenhouse for 48 h before EPG-DC recording, mortality bioassays, and RNA extraction.

### 2.3. Gene Expression Analysis after ASM Treatment

Pear leaf samples were collected from treated and control plants and immediately frozen at −80 °C. The total RNA was extracted using the Spectrum Plant Total RNA Prep kit (SIGMA Life Science, St. Louis, MO, USA) from 100 mg of tissue. Upon DNAse treatment (ThermoFisher Scientific, Waltham, MA, USA), 1 µg of RNA was retrotranscribed with a OneScript Plus cDNA Synthesis kit (Abm, Vancouver, BC, Canada). Real-time PCR reactions were performed in a CFX Connect Real-Time (Bio-Rad, Hercules, CA, USA) containing 1.6 µL of cDNA, 6 µL of SsoAdvanced Universal SYBR Green Supermix (Bio-Rad, Hercules, CA, USA), and 0.4 µL of each primer (10 μM) in a 12 µL final volume. The amplification conditions were 95° for 2 min, 40 cycles at 95 °C/10 s, and 60 °C/30 s. A melting curve analysis from 55 to 95 °C was applied after the amplification protocol. Gene expression was normalized using the *EF1α* reference gene and analysed by the Livak method [26] followed by the ANOVA nonparametric Mann-Whitney test. The primers used are shown in Table 1 (PCP sequences were obtained from the Genome Database for Rosaceae, GDR, [27]).

### 2.4. EPG-DC Recording

The EPG-DC recording was performed in the laboratory at 21 ± 1 °C under fluorescent light (4000 Lux, HF tubes). The EPG-DC recording involved 3rd instar nymphs as well as winter and summer forms of *C. pyri* adults (25 individuals for each stage) singularly placed on pear plants previously (48 h) treated with ASM or water (negative control). The EPG-DC system was composed of a 100-Hz Giga 4 amplifier (Wageningen University, Wageningen, The Netherlands) with 1-GΩ resistance input [28]. A thin gold electrode wire (diameter: 20 μm; length: 2 cm) was glued to the dorsum of each *C. pyri* individual using silver glue, as previously described [29]. The gold electrode was connected to the amplifier by a long and thick copper wire. A second electrode, made of copper, was deeply inserted in the wet soil of the potted pear plant.

During each registration, the plants, the insects, and the amplifier were placed inside a Faraday cage to avoid background electric noise. Each EPG-DC signal was recorded for 8 h and converted from analogic to digital (A–D) through an interface device (KPCI-3102, Keithley Instruments, Cleveland, OH, USA). If one insect fell from the leaf within the first hour of recording, the insect was repositioned once. Later falls were removed from the experiment. Falls were nevertheless quite rare (below 10% on average) At the end of the recording, dead insects were noted and excluded from further analyses. All waveforms were analysed and scored by the software Stylet+ (Wageningen University, Wageningen, The Netherlands) using the following characterization: Np: non probing; PA-PB: stylet penetration (sheath salivation); PC1: parenchyma penetration (sheath salivation); PC2: vascular parenchyma (unknown activity); PD: transition to phloem sieve elements; PE1: sieve element penetration (phloem salivation activity); PE2: sieve element penetration (phloem ingestion); PG: xylem vessel penetration (xylem ingestion) [29,30,31] (Supplementary Materials Figure S1). Once marked, all the recordings were individually selected for subsequent analysis. In particular, all recordings with electrical noises or bad electric connections were discarded, as suggested by Ripamonti and colleagues [32]. All waveforms recorded for both adults and nymphs were analysed by the ANOVA nonparametric Mann-Whitney test with the software package STATISTICA for Windows version 12 (StatSoft, Tulsa, OK, USA), in accordance with the nonsequential parameters [33].

### 2.5. Nymphs Mortality Bioassay

The foliar spray bioassays were performed as described [4], with slight modifications, in the laboratory at 23 ± 1 °C, 65% relative humidity (r.h.), and a 16:8 h LD photoperiod. Nine pear potted plants (cv. Abbé Fétel) were sprayed by a hand atomizer with 125 mg L^−1^ ASM and returned to the growth chamber for 48 h. Twenty 2nd instar reared *C. pyri* nymphs were gently placed on five leaves (4 nymphs per leaf) of each pear potted plant for a total of 9 replicates with 180 nymphs. A batch of nine pear plants sprayed only with distilled water was used as control. The surviving nymphs were scored 10 days after the placement. Nymphs were considered alive when they could walk and produce honeydew. The number of dead nymphs was calculated by subtracting the number of surviving nymphs from the total number of nymphs placed on the plants. Mortality data were analysed by the ANOVA nonparametric Mann-Whitney test with the software package STATISTICA for Windows version 12. The ASM efficacy (corrected mortality) was obtained by the Schneider-Orelli formula [34].

## 3. Results

### 3.1. Effect of ASM Treatment on Pear Pathogenesis-Related (PR) Proteins

**Figure 1:**
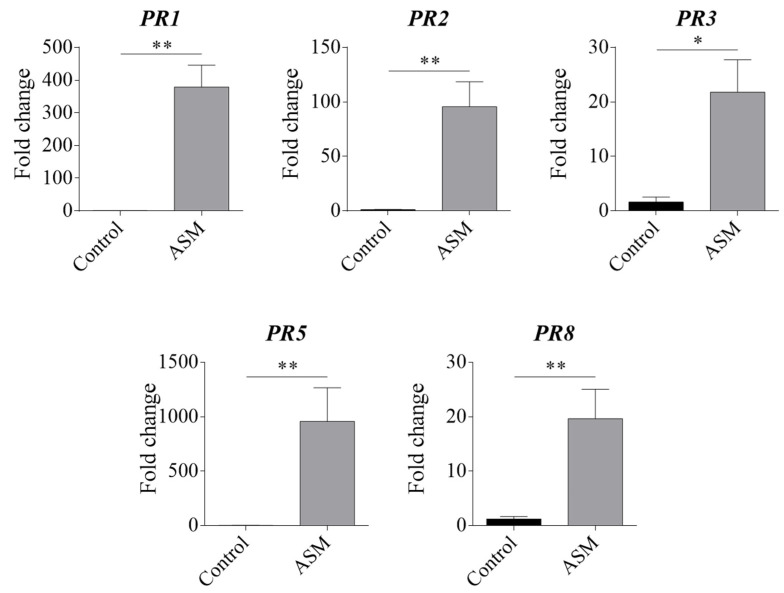
Fold-change values for *PR* genes upregulated 48 h after ASM application, normalized with the *EF1α* reference gene, and analysed by the Livak method. * *p* < 0.05, ** *p*< 0.01 vs. control according to the Mann-Whitney test.

Pear plants, 48 h after ASM application, were firstly tested at the molecular level to confirm the elicitor effect. RT-qPCR analyses performed on RNA extracted from ASM- or water-treated pear leaves confirmed that several *PR* genes (*PR1*, *PR2*, *PR3*, *PR5*, and *PR8*, known from the literature to be induced by ASM-application in pear and other plant species [12,13,14]) were strongly upregulated by ASM, as expected, when normalized to the *EF1α* reference gene (Figure 1).

### 3.2. EPG-DC Recording

The EPG-DC recordings were performed to assess the effect of ASM on *C. pyri* probing behaviour. Probing was registered for 8 h on insects positioned on leaves 48 h after ASM or water applications. Table 2 shows that, for nymphs, the events of stylet penetration in epidermal and parenchymal tissues (along with sheath salivation) (PA, PB, and PC1 [29]) were more frequent (18.82 events/insect vs. 12.55 events/insect, *p* = 0.04) and shorter on ASM-treated plants as compared to the control (10.07 min/insect vs. 15.60 min/insect, *p* = 0.007). Once the nymph’s stylet reached the phloem, ASM treatment induced a statistically significant difference in the total duration of phloem salivation events (PE1 [29]) (41.62 min/insect vs. 16.20 min/insect, *p* = 0.01) and in the number of phloem salivation events (PE1, 10.09 events/insect vs. 4.86 events/insect, *p* = 0.003), while the duration of each phloem salivation event did not vary between the treated and control plants. Similar differences were also detected in the events related to transition to phloem (PD [29]). Other events, such as the phloem ingestion (PE2 [29]), were apparently not affected by the ASM application during the 8 h of EPG-DC recording with nymphs. On the other hand, no significant differences were observed in all parameters measured when *C. pyri* adults (summer and winter forms) were used in the feeding test on ASM-treated or control plants (Table 3 and Table 4).

### 3.3. Nymph Mortality Bioassay

The percentage of mortality of *C. pyri* 2nd instar nymphs feeding on ASM- or water- (control) treated pear plants is reported in Table 5. The mortality on ASM-treated plants was statistically different from the water-treated control (27.97 vs. 16.30; *p* = 0.009), but the ASM efficacy, expressed as corrected mortality, was nevertheless very weak (13.94%).

## 4. Discussion

RT-qPCR analysis confirmed that ASM was able to activate plant defences in pear plantlets. In fact, all the PR protein encoding genes tested were strongly upregulated 48 h after treatment. These data are in agreement with the ASM-induced gene upregulation described in several works in many species [13], including apple [14] and pear [12].

Nevertheless, the EPG results indicated that ASM application only weakly interfered with *C. pyri* nymph probing activities. In particular, ASM caused a significant increase in the mean number of stylet penetration events in nonvascular tissues and in salivation events into the phloem, once the stylet reached this tissue. Furthermore, these effects were only visible in nymphs, which were known to have a probing behaviour mainly limited to phloem [1,29,30]. Winter or summer adults’ behaviour, which is known to be less frequent in phloem [30], was unaffected by the ASM application. A previous study [25] revealed that tomato plants were less acceptable to *M. persicae* upon ASM application, inducing a sharp decrease in the total duration and in the number of phloem ingestions and a significant reduction in aphid fecundity [23]. These results on tomato after ASM application, as suggested by [24], might be linked to the induction of the defensive pathways, which could also protect susceptible tomato cultivars against aphid infestation.

The modest modifications of *C. pyri* nymphs feeding on pear plants after ASM application can explain and confirm the data obtained on *C. pyricola* [9,11]. ASM was able to activate a systemic defence response in pear trees causing a reduction in *C. pyricola* oviposition and in nymph survival, which led to a general but weak reduction in psylla population. These effects were observed in different conditions, from the laboratory to the managed or unmanaged orchards, after one or multiple ASM applications. The modest activity of ASM on *C. pyri* feeding as well as on *C. pyricola* nymphs’ survival do not warrant the use of ASM alone for the control of psyllids. However, elicitors are often used to manage pear fire blight, therefore their use might also contribute to control pear psylla populations within integrated pest management approaches. Furthermore, one cannot exclude that ASM might have an effect on the pathogen transmitted by pysllas such as the pear decline “*Candidatus* Phytoplasma pyri”; unfortunately, no specific work has been conducted. On the other hand, the effect of plant elicitors, such as ASM, on the resistance to a wide range of pathogens including other phytoplasms is well known [35,36,37,38]. More studies will be required to evaluate the true role of ASM in overall pear protection management.

## Figures and Tables

**Table 1:** Primers used in this study. PR: Pathogenesis-Related proteins; EF1α: Elongation Factor 1 alpha.

Gene	Forward Primer (5′-3′)	Reverse Primer (5′-3′)	Accession nr
*PR1*	GCACAAAACTACGCCAACCA	CCTTTCCATCAGCACACGAG	JQ965001
*PR2*	TCTCCTGCCTGCCATCCAAA	CCCACTGTTGACGAGGAAGT	PCP025709
*PR3*	ACCTAGACGGCGCTATCCAA	CCATTGGCTCCAACTGTTCA	JQ965010
*PR5*	GCAGTTCCACCAGCAACTTTA	TTAACATTGGCAGGGCACGAT	PCP037574
*PR8*	ACCCAGGTGTTCATGGGGTTA	CCGTTGTCATAGAACCTGCTC	PCP012163
*EF1α*	ACAAGATGGATGCCACCACTC	AGGTTGGTGGACCTCTCAATC	PCP028497

**Table 2:** Comparison of EPG-DC waveforms (mean time in minutes ± standard error of the mean, SEM, or mean number of events ± SEM) (8 h of recording) of *C. pyri* nymphs (*n* = 22 on ASM, *n* = 22 control) feeding on ASM- or water- (control) treated pear plants. Waveforms were classified according to [29,30,31]. * *p* < 0.05 vs. control according to the Mann-Whitney test.

Total Duration in Minutes/Insect							
	**Non-probing**	**PA, PB, PC1**	**Xylem ingestion (PG)**	**PC2**	**PD**	**Phloem salivation (PE1)**	**Phloem ingestion (E2)**
ASM	105.66 ± 15.02	161.36 ± 14.89	9.08 ± 3.75	2.49 ± 2.10	3.25 ± 0.57	41.62 ± 9.81	156.51 ± 27.65
Control	134.32 ± 21.31	164.96 ± 16.60	23.83 ± 6.68	3.22 ± 2.00	1.53 ± 0.31	16.20 ± 3.11	135.89 ± 25.45
*p* value	0.36	0.93	0.13	0.60	0.005 *	0.01 *	0.73
**Number of Events per Insect**							
	**Non-probing**	**PA, PB, PC1**	**Xylem ingestion (PG)**	**PC2**	**PD**	**Phloem salivation (PE1)**	**Phloem ingestion (E2)**
ASM	9.41 ± 1.45	18.82 ± 2.18	0.32 ± 0.12	0.41 ± 0.32	9.73 ± 1.63	10.09 ± 1.66	2.14 ± 0.34
Control	7.82 ± 1.15	12.55 ± 1.35	0.86 ± 0.22	0.23 ± 0.09	4.95 ± 0.95	4.86 ± 0.95	1.77 ± 0.41
*p* value	0.47	0.04 *	0.07	0.65	0.006 *	0.003 *	0.26
**Duration of Event in Minutes/Insect**							
	**Non-probing**	**PA, PB, C1**	**Xylem ingestion (PG)**	**PC2**	**PD**	**Phloem salivation (PE1)**	**Phloem ingestion (PE2)**
ASM	14.99 ± 3.46	10.07 ± 1.17	8.05 ± 3.40	0.69 ± 0.40	0.32 ± 0.03	3.45 ± 0.64	74.23 ± 23.67
Control	24.41 ± 5.60	15.60 ± 2.16	16.12 ± 4.43	3.22 ± 2.00	0.30 ± 0.02	4.20 ± 0.87	100.31 ± 22.37
*p* value	0.21	0.007 *	0.18	0.55	1	1	1

**Table 3:** Comparison of EPG-DC waveforms (mean time in minutes ± standard error of the mean, SEM, or mean number of events ± SEM) (8 h of recording) of *C. pyri* summer adults (*n* = 16 on ASM, *n* = 14 control) feeding on ASM- or water- (control) treated pear plants. Waveforms were classified according to [29,30,31].

			Total Duration in Minutes/Insect				
	**Non-probing**	**PA, PB, PC1**	**Xylem ingestion (PG)**	**PC2**	**PD**	**Phloem salivation (PE1)**	**Phloem ingestion (E2)**
ASM	134.68 ± 28.91	173.16 ± 23.38	27.17 ± 17.21	40.33 ± 29.02	3.14 ± 1.35	38.29 ± 19.00	63.23 ± 33.17
Control	186.16 ± 23.93	127.73 ± 19.23	44.23 ± 18.21	8.91 ± 4.71	1.46 ± 0.35	43.13 ± 14.43	68.38 ± 28.01
*p* value	0.23	0.20	0.65	1	0.23	0.96	0.77
	**Number of Events per Insect**						
	**Non-probing**	**PA, PB, PC1**	**Xylem ingestion (PG)**	**PC2**	**PD**	**Phloem salivation (PE1)**	**Phloem ingestion (E2)**
ASM	13.50 ± 2.25	24.83 ± 4.94	0.67 ± 0.33	3.17 ± 2.01	12.17 ± 5.37	12.17 ± 5.37	2.50 ± 0.67
Control	15.43 ± 3.44	21.79 ± 2.89	1.07 ± 0.29	1.79 ± 0.59	6.21 ± 1.42	6.36 ± 1.49	1.93 ± 0.56
*p* value	0.90	0.59	0.50	1	0.40	0.43	0.30
	**Duration of Event in Minutes/Insect**						
	**Non-probing**	**PA, PB, PC1**	**Xylem ingestion (PG)**	**PC2**	**PD**	**Phloem salivation (PE1)**	**Phloem ingestion (E2)**
ASM	10.70 ± 2.14	7.83 ± 1.57	24.31 ± 17.21	4.61 ± 2.62	0.34 ± 0.08	3.54 ± 0.91	42.66 ± 25.97
Control	17.43 ± 3.79	6.11 ± 0.82	22.03 ± 7.74	2.48 ± 0.77	0.24 ± 0.04	4.94 ± 1.02	38.49 ± 23.40
*p* value	0.34	0.34	0.83	0.86	0.10	0.43	0.96

**Table 4:** Comparison of EPG-DC waveforms (mean time in minutes ± standard error of the mean, SEM, or mean number of events ± SEM) (8 h of recording) of *C. pyri* winter adults (*n* = 20 on ASM, *n* = 22 control) feeding on ASM- or water- (control) treated pear plants. Waveforms were classified according to [29,30,31].

	**Total Duration in Minutes/Insect**						
	**Non-probing**	**PA, PB, PC1**	**Xylem ingestion (PG)**	**PC2**	**PD**	**Phloem salivation (PE1)**	**Phloem ingestion (E2)**
ASM	172.97 ± 21.13	113.40 ± 14.43	115.52 ± 13.43	62.42 ± 15.95	1.815 ± 0.56	41.41 ± 3.28	68.82 ± 41.92
Control	146.10 ± 22.20	131.13 ± 16.07	130.92 ± 20.50	81.94 ± 17.56	5.02 ± 3.94	21.79 ± 8.96	23.05 ± 16.77
*p* value	0.46	0.42	0.96	0.56	0.72	0.65	0.46
	**Number of Events per Insect**						
	**Non-probing**	**PA, PB, PC1**	**Xylem ingestion (PG)**	**PC2**	**PD**	**Phloem salivation (PE1)**	**Phloem ingestion (E2)**
ASM	10.65 ± 1.79	14.65 ± 2.08	2.20 ± 0.39	2.80 ± 0.83	3.00 ± 1.25	2.15 ± 0.83	0.95 ± 0.45
Control	11.18 ± 1.72	14.63 ± 2.13	2.45 ± 0.29	4.77 ± 1.29	1.50 ± 0.46	1.50 ± 0.44	0.63 ± 0.25
*p* value	0.91	1	0.44	0.15	0.94	0.87	0.84
	**Duration of Event in Minutes/Insect**						
	**Non-probing**	**PA, PB, PC1**	**Xylem ingestion (PG)**	**PC2**	**PD**	**Phloem salivation (PE1)**	**Phloem ingestion (E2)**
ASM	35.05 ± 10.24	9.42 ± 1.16	55.28 ± 10.46	17.48 ± 3.74	0.22 ± 0.03	2.70 ± 0.85	12.14 ± 8.40
Control	19.70 ± 4.60	10.56 ± 1.45	49.31 ± 4.42	14.43 ± 2.34	0.95 ± 0.64	3.45 ± 0.87	20.44 ± 17.15
*p* value	0.19	0.42	0.80	0.68	0.14	0.68	0.56

**Table 5:** Mortality data of *C. pyri* nymphs on ASM-sprayed pear plants (*N* = 9) compared to control plants (*N* = 9) and percentage of ASM efficacy (corrected mortality) calculated according to [34]. ** *p* < 0.01 vs. control according to the Mann-Whitney test.

	N	Total *C. pyri* Nymphs per Treatment	Mortality % (±SEM)	% ASM Efficacy (Corrected Mortality)
ASM	9	180	27.97 ± 2.44	13.94
Control	9	180	16.30 ± 2.77	-
*p* value			0.009 **	

## Data Availability

The datasets generated and analysed during the current study are available within the article, as well as from the corresponding author on reasonable request.

## Supplementary Materials

The following supporting information can be downloaded at: https://www.mdpi.com/article/10.3390/insects13060525/s1, Figure S1: Schematical representation of typical *C. pyri* probing waveforms for winter- and summer-adults and nymphs.
