# Supplementary material for: Effects of Acibenzolar-S-methyl on the Probing Behaviour and Mortality of Cacopsylla pyri on Pear Plants
Source: Insects. 2022 Jun 6;13(6):525. doi: 10.3390/insects13060525 (PMC9225062; doi:10.3390/insects13060525)
Supplement: Supplementary file 1 [file insects-13-00525-s001.zip › insects-1686440-supplementary.pdf]

insects

MDPI

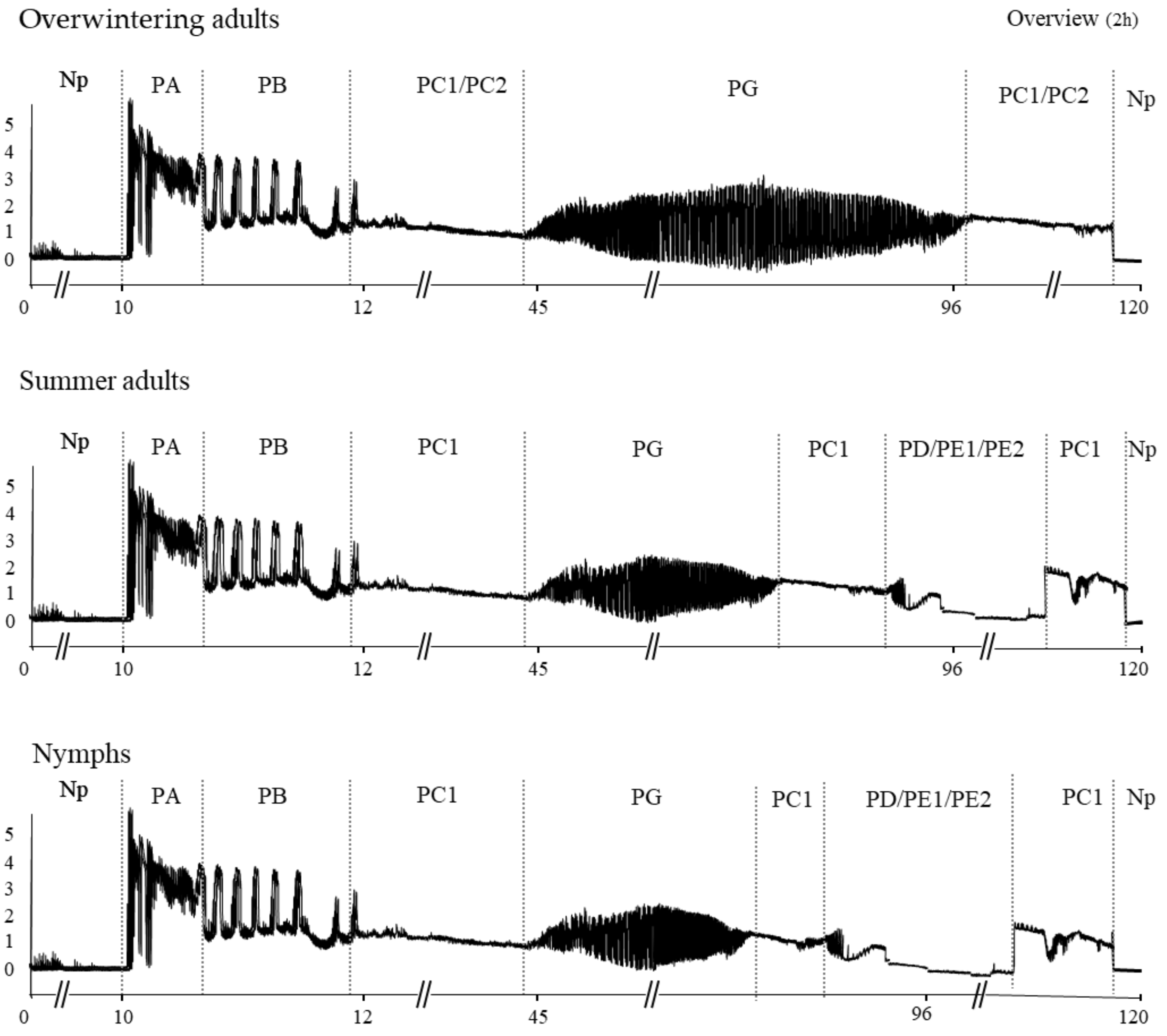

Supplementary Figure S1. Schematical representation of typical *C. pyri* probing waveforms for winter- and summer-adults and nymphs. Np: non probing; PA-PB: stylet penetration (sheath salivation); PC1: Parenchyma penetration (sheath salivation); PC2: Vascular parenchyma (unknown activity); PD: Transition to phloem sieve elements; PE1: Sieve element penetration (phloem salivation activity); PE2: Sieve element penetration (phloem ingestion); PG: Xylem vessel penetration (xylem ingestion) [29-31].
